# Different Genes Interact with Particulate Matter and Tobacco Smoke Exposure in Affecting Lung Function Decline in the General Population

**DOI:** 10.1371/journal.pone.0040175

**Published:** 2012-07-06

**Authors:** Ivan Curjuric, Medea Imboden, Rachel Nadif, Ashish Kumar, Christian Schindler, Margot Haun, Florian Kronenberg, Nino Künzli, Harish Phuleria, Dirkje S. Postma, Erich W. Russi, Thierry Rochat, Florence Demenais, Nicole M. Probst-Hensch

**Affiliations:** 1 Department of Epidemiology and Public Health, Swiss Tropical and Public Health Institute SwissTPH, Basel, Switzerland; 2 University of Basel, Basel, Switzerland; 3 INSERM, U1018, CESP Centre for research in Epidemiology and Population Health, Respiratory and Environmental Epidemiology Team, Villejuif, France; 4 Université Paris-Sud 11, UMRS 1018, Villejuif, France; 5 Wellcome Trust Centre for Human Genetics, University of Oxford, Oxford, United Kingdom; 6 Division of Genetic Epidemiology, Department of Medical Genetics, Molecular and Clinical Pharmacology, Innsbruck Medical University, Innsbruck, Austria; 7 Department of Pulmonary Medicine and Tuberculosis, University Medical Center Groningen, University of Groningen, Groningen, The Netherlands; 8 Division of Pulmonary Medicine, University Hospital Zürich, Zürich, Switzerland; 9 Division of Pulmonary Medicine, Geneva University Hospitals, Geneva, Switzerland; 10 INSERM, U946, Genetic Variation and Human Diseases Unit, Paris, France; 11 Fondation Jean Dausset - Centre d’Etude du Polymorphisme Humain (CEPH), Paris, France; 12 Université Paris Diderot, Sorbonne Paris Cité, Institut Universitaire d’Hématologie, Paris, France; Universite de Montreal, Canada

## Abstract

**Background:**

Oxidative stress related genes modify the effects of ambient air pollution or tobacco smoking on lung function decline. The impact of interactions might be substantial, but previous studies mostly focused on main effects of single genes.

**Objectives:**

We studied the interaction of both exposures with a broad set of oxidative-stress related candidate genes and pathways on lung function decline and contrasted interactions between exposures.

**Methods:**

For 12679 single nucleotide polymorphisms (SNPs), change in forced expiratory volume in one second (FEV_1_), FEV_1_ over forced vital capacity (FEV_1_/FVC), and mean forced expiratory flow between 25 and 75% of the FVC (FEF_25-75_) was regressed on interval exposure to particulate matter <10 µm in diameter (PM10) or packyears smoked (a), additive SNP effects (b), and interaction terms between (a) and (b) in 669 adults with GWAS data. Interaction p-values for 152 genes and 14 pathways were calculated by the adaptive rank truncation product (ARTP) method, and compared between exposures. Interaction effect sizes were contrasted for the strongest SNPs of nominally significant genes (p_interaction_<0.05). Replication was attempted for SNPs with MAF>10% in 3320 SAPALDIA participants without GWAS.

**Results:**

On the SNP-level, rs2035268 in gene SNCA accelerated FEV_1_/FVC decline by 3.8% (p_interaction_ = 2.5×10^−6^), and rs12190800 in PARK2 attenuated FEV1 decline by 95.1 ml p_interaction_ = 9.7×10^−8^) over 11 years, while interacting with PM10. Genes and pathways nominally interacting with PM10 and packyears exposure differed substantially. Gene CRISP2 presented a significant interaction with PM10 (p_interaction_ = 3.0×10^−4^) on FEV_1_/FVC decline. Pathway interactions were weak. Replications for the strongest SNPs in PARK2 and CRISP2 were not successful.

**Conclusions:**

Consistent with a stratified response to increasing oxidative stress, different genes and pathways potentially mediate PM10 and tobacco smoke effects on lung function decline. Ignoring environmental exposures would miss these patterns, but achieving sufficient sample size and comparability across study samples is challenging.

## Introduction

Lung function is an important determinant of respiratory health and life expectancy [1], [2], [3], [4]. Its longitudinal course is affected by different environmental exposures such as active tobacco smoking, environmental tobacco smoke exposure [5], possibly workplace exposures to dusts and fumes [6], [7], [8], [9] as well as ambient air pollution [10]. Both air pollution and tobacco smoke are known to contain free radicals and to induce their direct formation at the tissue level causing damage of cell walls, proteins and DNA, and chronic tissue inflammation and remodeling in the long run [11], [12]. Upon exposure, different protein systems including those scavenging reactive oxygen species (ROS) are up-regulated, and the level of response is influenced by variation in underlying genes. Likewise, polymorphisms in oxidative stress related candidate genes like *gluthathione s-transferases (GSTs)*, *microsomal epoxide hydroxylase (EPHX)*, or *heme-oxygenase 1 (HMOX-1),* have been associated with lung function decline and chronic obstructive pulmonary disease (COPD), a disease characterized by accelerated, progressive lung function loss [13], [14], [15], [16]. But most of these candidate genes have not been consistently replicated across studies and populations according to a recent review [15]. Similarly, genome-wide association studies (GWAS) of lung function partially struggled with replication [17], [18]. Further, in GWAS on lung function level or COPD prevalence [17], [18], [19], [20], [21], [22] association signals in known oxidative-stress genes were not strong [23].

Reasons for non-replication could be genetic heterogeneity across populations, or also sub-phenotypes of disease [24]. However, it is also possible that differences in environmental factors, and hence presence of gene-environment interaction play a role. To the best of our knowledge, only one published genome-wide interaction study examining the effect of farming exposure on childhood asthma has taken into account gene-environment interaction in respiratory disease to date [25]. This gap in the scientific literature is probably due to increased sample size requirements when assessing gene-environment interactions with classical analysis methods [26], [27]. However, their importance in respiratory disease has previously been shown in candidate gene studies focusing on single genes and SNPs therein [28], [29], [30], as well as follow-up studies of GWAS [31], [32].

Analysis methods such as pathway- or gene-set analyses [33] can at least partly overcome sample size restrictions by reducing the dimensionality of the data, and thus offer a promising alternative study approach. Based on biological knowledge of genes and their organization into molecular pathways, the longitudinal course of lung function might be better explained by accumulating interaction signals between environmental exposures and multiple SNPs of the same gene, or different genes involved in the same canonical pathway contributing to a functional entity in the organism.

We thus aimed to investigate to which extent oxidative-stress related genes and pathways interact significantly with interval exposure to ambient particulate matter of mean diameter <10 µm (PM10) or active tobacco smoking on natural lung function decline using genome-wide data from non-asthmatic adults of the Swiss Study on Air Pollution and Lung and Heart Diseases in Adults (SAPALDIA). SNP-level interaction signals were integrated onto upper biological levels to identify significantly interacting genes and pathways. The impact of PM10 exposure on lung function decline was contrasted to tobacco smoking by comparing patterns of associations at the gene- and pathway level, as well as interaction effect sizes for the strongest interacting SNP within genes.

## Methods

### Ethics Statement

All participants gave written informed consent. The study was approved by the Overall Regional Ethics Commission for Clinical Medicine (Swiss Academy of Medical Sciences, Basel, Switzerland) and the responsible cantonal ethics committees of each study centre (Ethics Commissions of the cantons Aarau, Basel, Geneva, Grisons, Ticino, Valais, Vaud, and Zürich).

### Study Population

SAPALDIA is a population-based cohort study established in 1991 to assess the effects of long-term exposure to ambient air pollution on respiratory health, with a first follow-up examination in 2002. Participants were residents from 8 communities throughout Switzerland aged 18–60 years at baseline. Details of the study design and methodology were published elsewhere [10], [34], [35].

The current work is based on up to 669 non-asthmatic participants with genome-wide data fulfilling quality control criteria and complete data on sex, age, height, PM10- and smoking exposure (**see Figure S1**). Participants without genome-wide data served as replication sample.

### Spirometric Measurements

Spirometry was performed without bronchodilation. Identical spirometry protocols and devices (Sensormedics model 2200, Yorba Linda, USA) were used in 1991 and 2002 [36], [37]. Participants were in an upright sitting position and performed three to eight forced expiratory lung function maneuvers according to American Thoracic Society quality criteria [38]. At least two acceptable measurements of forced vital capacity (FVC) and forced expiratory volume in the first second (FEV_1_) were obtained. Forced expiratory flow between 25 and 75% of the FVC (FEF_25–75_) was recorded.

In the present study we studied the decline of FEV_1_, the ratio FEV_1_/FVC and FEF_25–75_ between 1991 and 2002, as measures of airway obstruction, calculated by subtracting the first measurement from the second (measurement at SAPALDIA2– measurement at SAPALDIA1).

### Health Questionnaire Data

Smoking information was assessed by questionnaire. At each examination, never smokers were defined as having smoked less than 20 packs of cigarettes or 360 g of tobacco in their life, ex-smokers as having quit smoking at least 30 days before the interview, and current smokers as those who reported active smoking [39]. Packyears smoked between baseline and follow-up examination were used for comparison with interval PM10 exposure, and were calculated by dividing the number of cigarettes per day by 20 (giving number of cigarette packs) and multiplying the result with years of exposure.

### Air pollution Exposure

Similarly to calculating packyears, interval PM10 exposure was defined by summing individual average home outdoor exposure to PM10 over each year of follow-up, giving estimates in (µg/m^3^) * years. Annual average exposures were calculated by using exposure estimates from Gaussian Dispersion models on a 200m×200m grid throughout Switzerland for years 1990 and 2000, and interpolating historical trends from fixed air pollution monitoring stations. Participants were assigned individual annual exposure estimates via their geo-referenced residence addresses, taking account of residence changes during follow-up. Details on exposure modeling are given elsewhere [40].

### SNP Genotyping and Imputation

Blood for DNA-analysis was drawn in 2002 in participants giving consent to genetic analyses [34].

Genome-wide genotyping was done on the Illumina Human 610quad BeadChip in the framework of the EU-funded GABRIEL study [41], a large consortium aiming to uncover genetic and environmental causes of asthma. The current work focused on the non-asthmatic portion of participants.

567′589 successfully genotyped autosomal SNPs were imputed to 2.5 Mio using MACH v 1.0 software [42] and the HapMap v22 CEPH reference panel of Utah residents with ancestry from northern and western Europe [43].

Strict quality control (QC) was applied by excluding samples with <97% genotyping success rate, non-European origin, cryptic relatedness or sex-inconsistencies, as well as SNPs with Hardy-Weinberg equilibrium p-value<10^−4^, call rate <97%, minor allele frequency (MAF) <5% or low imputation quality (Rsq<0.5). A total of 2′168′681 SNPs withstood QC, and genome-wide data was finally available in 669 non-asthmatic individuals with environmental exposure data.

Replication genotyping was attempted for two interacting SNPs (rs360563 in gene *CRISP2*, and rs12190800 in *PARK2*) with MAF>10%. Genotyping was done using the iPLEX Gold MassARRAY (SEQUENOM, San Diego, USA) on the whole SAPALDIA study population including the analysis sample, as the costs for manual sample selection outweigh those of additional genotyping. The replication sample consisted of 3320 successfully genotyped participants with complete data for covariates and all three lung function parameters (**see Figure S1**).

### Definition of Oxidative-stress Genes and Pathways

Oxidative stress related genes were defined as either coding proteins that directly scavenge or endogenously produce ROS, their immediate regulators, or key genes in cascades triggered by oxidative stress. They were identified by searching the Gene Ontology database [44] with the term “response to oxidative stress” and GeneCards with “oxidative stress” in the pathway field of the advanced search option (http://www.genecards.org/index.php?path=/Search/Advanced/, accessed November 2010). Resulting gene lists were further enriched by literature reviews [45], [46], [47], [48], [49]. By feeding the gene lists into Ingenuity Pathway Analysis (Ingenuity® Systems, www.ingenuity.com), 14 molecular pathways related to oxidative stress and environmental exposures of interest were identified (**Table 1**).

**Table 1:** Mapping of candidate oxidative-stress genes to molecular pathways of interest.

**PATHWAY**	**GENES**
**Arachidonic Acid Metabolism**	ALOX12 CYP1A1 CYP1A2 DHRS2 EPHX2 GPX1 GPX2 GPX3 GPX4 GPX5 GPX6 GPX7 GPX8 GSTK1 GSTT1 GSTZ1 MGST2 MGST3 PLA2G4A PRDX6 PTGS1 PTGS2
**Aryl Hydrocarbon Receptor Signaling**	ARNT CDKN1A CYP1A1 CYP1A2 EP300 FOS GSTK1 GSTM1 GSTM2 GSTM3 GSTM4 GSTM5 GSTO1 GSTO2 GSTP1 GSTT1 GSTT2 JUN MGST1 MGST2 MGST3 NFE2L2 NFKB1 NQO1 NQO2 RELA TP53
**fMLP Signaling in Neutrophils**	MAP2K1 NCF2 NFKB1 NOX3 NOX4 PLCB1 PRKCA RAC1 RELA
**Glutathione Metabolism**	GCLC GCLM GLRX GPX1 GPX2 GPX3 GPX4 GPX5 GPX6 GPX7 GPX8 GSR GSS GSTK1 GSTM1 GSTM2 GSTM3 GSTM4 GSTM5 GSTO1 GSTO2 GSTP1 GSTT1 GSTT2 GSTZ1 IDH1 MGST1 MGST2 MGST3 PRDX6
**IL-6 Signaling**	CHUK COL1A1 FOS GRB2 JAK2 JUN MAP2K1 MAPK14 NFKB1 RELA
**Metabolism of Xenobiotics by Cytochrome P450**	AKR1A1 CYP1A1 CYP1A2 DHRS2 EPHX1 GSTK1 GSTM1 GSTM2 GSTM3 GSTM4 GSTM5 GSTO1 GSTO2 GSTP1 GSTT1 GSTT2 GSTZ1 MGST1 MGST2 MGST3
**Methane Metabolism**	CAT EPX LPO MPO PRDX1 PRDX2 PRDX5 PRDX6 TPO
**Mitochondrial Dysfunction**	CAT GLRX2 GPX4 GPX7 GSR NDUFA12 NDUFA13 NDUFA6 NDUFS1 NDUFS2 NDUFS3 NDUFS4 NDUFS8 PARK2 PARK7 PRDX3 PRDX5 PSEN1 SNCA SOD2 TXN2 TXNRD2 UCP2
**NF-κB Signaling**	CHUK EGFR EP300 INSR NFKB1 RAC1 RAC2 RELA RIPK1 TGFBR2 TLR4
**NRF2-mediated Oxidative Stress Response**	ABCC1 AKR1A1 AKR7A2 AKR7A3 AOX1 CAT EP300 EPHX1 FOS FOSL1 GCLC GCLM GPX2 GSR GSTK1 GSTM1 GSTM2 GSTM3 GSTM4 GSTM5 GSTO1 GSTO2 GSTP1 GSTT1 GSTT2 HMOX1 JUN KEAP1 MAP2K1 MAPK14 MGST1 MGST2 MGST3 NFE2L2 NQO1 NQO2 PRDX1 PRKCA SOD1 SOD2 SOD3 TXN TXNRD1
**Oxidative** **Phosphorylation**	NDUFA12 NDUFA13 NDUFA6 NDUFS1 NDUFS2 NDUFS3 NDUFS4 NDUFS8
**Production of Nitric Oxide and Reactive Oxygen Species in macrophages**	CAT CHUK CYBA FOS JAK2 JUN MAP2K1 MAPK14 MPO NCF2 NFKB1 NOS2 PLCG1 PPP2CB PRKCA RAC1 RAC2 RELA STAT1 TLR4
**Xenobiotic Metabolism Signaling**	ARNT CAT CYP1A1 CYP1A2 EP300 FMO2 GCLC GSTK1 GSTM1 GSTM2 GSTM3 GSTM4 GSTM5 GSTO1 GSTO2 GSTP1 GSTT1 GSTT2 HMOX1 KEAP1 MAP2K1 MAPK14 MGST1 MGST2 MGST3 NFE2L2 NFKB1 NOS2 NQO1 NQO2 PPP2CB PRKCA RELA SOD3
**NOT MAPPED TO PATHWAY**	AATF AGT AGTR1 ATOX1 CCL5 CP CRISP2 CYGB DHCR24 DUSP1 ERCC1 GLRX3 GLRX5 GSTCD HMOX2 HP MSRA MT2A NAPRT1 NOS1 NOS3 NOX5 NOXO1 OGG1 OXR1 PNKP PSMB5 PTK2B PXDN PYCR1 SCARA3 SEPP1 SLC23A2 SRXN1 STK25 TXNIP

Gene regions were defined by retrieving transcription start and end positions in the ‘gene track’ of the UCSC browser (http://genome.ucsc.edu/) [50], genome build 18 (March 2006), and adding 20 kilo-bases to each end. Referring to dbSNP version 126, available SNP data was matched to gene regions. Data was available for 152 autosomal genes (**Table 1**), of which 46 mapped once to a pathway, 33 twice, and 37 three times or more. Thirty-six genes did not map to one of the 14 pathways, but were related to oxidative stress based on their function. Details on gene size, SNP-coverage and pathway mapping are given in Table S1 (**see Table S1**). Gene specific allele dosage files in MACH format were used for analysis.

### Statistical Analysis

#### Characterization of study population

The distribution of sex, age, baseline lung function parameters, their change during follow-up as well as packyears exposure during follow-up was tabulated according to categories of smoking status (never, former and current smokers) and interval PM10 exposure (high versus low exposure, defined by the median value) (Table 2). To assess a potential impact of loss to follow-up on our results, our study population consisting of up to 669 non-asthmatic adults with high quality genome-wide data and complete information on model covariates was compared to non-asthmatic participants examined at follow-up without genome-wide data (n = 3833), and to those completing only baseline examination (n = 1299) by means of descriptive tables and tests for independent samples (see Table S2).

**Table 2:** Distribution of main characteristics by smoking status at follow-up and PM_10_ exposure during follow-up (N = 650^1^).

		Interval PM_10_ EXPOSURE								
		(median:239.0 µg/m^3^*years)								
SMOKING CATEGORY		exposure < median					exposure≥ median			
	variable	*value* 2	*range*				*value* 2	*range*		
**Never**	n	**152**					**145**			
**Smoker**	female sex [%]	**67.1**					**53.8**			
	age at follow-up [years]	**53.0**		31.1	–	71.5	**52.7**	29.8	–	71.8
	FEV1 [L]	**3.4**		2.0	–	5.2	**3.6**	2.4	–	6.0
	FEV1 decline [L]	**−0.3**		**−**1.1	–	1.0	**−0.3**	**−**1.5	–	0.5
	FEV_1_/FVC [%]	**79.2**		61.4	–	98.0	**81.0**	62.5	–	99.8
	FEV_1_/FVC decline [%]	**−3.9**		**−**13.2	–	9.4	**−4.4**	**−**21.6	–	8.2
	FEF_25–75_ [L/sec]	**3.2**		1.3	–	7.5	**3.6**	1.2	–	6.8
	FEF_25–75_ decl. [L/sec]	**−0.7**		**−**2.5	–	2.1	**−0.8**	**−**3.7	–	0.6
	pack years d. follow-up	**n.a.**					**n.a.**			
**Former**	n	**98**					**102**			
**Smoker**	female sex [%]	**40.8**					**52.9**			
	age at follow-up [years]	**54.1**		32.7	–	72.0	**54.4**	30.8	–	71.9
	FEV1 [L]	**3.8**		2.2	–	5.4	**3.6**	2.3	–	5.6
	FEV1 decline [L]	**−0.4**		**−**1.2	–	0.3	**−0.4**	**−**1.4	–	0.6
	FEV_1_/FVC [%]	**78.8**		60.0	–	97.3	**80.6**	66.4	–	95.2
	FEV_1_/FVC decline [%]	**−3.2**		**−**16.1	–	12.4	**−4.7**	**−**20.7	–	13.7
	FEF_25–75_ [L/sec]	**3.6**		1.4	–	7.7	**3.6**	1.5	–	7.2
	FEF_25–75_ decl. [L/sec]	**−0.8**		**−**2.8	–	1.7	**−0.8**	**−**3.6	–	1.6
	pack years d. follow-up	**0.0**		0.0	–	25.0	**0.0**	0.0	–	35.0
**Current**	n	**75**					**78**			
**Smoker**	female sex [%]	**37.3**					**46.2**			
	age at follow-up [years]	**52.8**		29.8	–	70.8	**49.7**	30.3	–	70.6
	FEV1 [L]	**3.6**		2.4	–	5.7	**3.7**	1.8	–	6.8
	FEV1 decline [L]	**−0.5**		**−**1.5	–	0.1	**−0.4**	**−**1.3	–	0.3
	FEV_1_/FVC [%]	**77.6**		59.3	–	94.5	**79.0**	49.0	–	97.1
	FEV_1_/FVC decline [%]	**−6.3**		**−**20.5	–	3.9	**−4.9**	**−**21.5	–	7.9
	FEF_25–75_ [L/sec]	**3.3**		1.4	–	7.4	**3.5**	0.7	–	7.3
	FEF_25–75_ decl. [L/sec]	**−1.0**		**−**3.0	–	0.4	**−0.8**	**−**2.4	–	0.7
	pack years d. follow-up	**10.9**		0.0	–	27.3	**9.0**	0.0	–	24.0

1 restricted to sample with complete data on all three lung function parameters. Regarding FEV_1_, sample size with complete covariate data would be n = 669.

2 means for age and lung function values, medians for pack years exposures.

#### Gene- and pathway-environment interaction analysis

The interaction of genetic variation and exposure to PM10 or tobacco smoke on lung function decline was assessed in different stages.

First, SNP level analyses on decline in FEV1, FEV1/FVC and FEF25–75 were done for each gene separately using multiple linear regression in ProbABEL v0.1.3 (http://www.genabel.org) [51] with robust sandwich-estimation of standard errors. Models specified an additive SNP-effect, main effects for packyears smoked and interval PM10 exposure between surveys, and an interaction term between the SNP-variable and either exposure. They adjusted for sex, age and height at follow-up, packyears smoked up to baseline, principal components of population ancestry, and study area. No adjustment for ageing during follow-up was made, since follow-up time was 11 years for all participants. Complete data including covariates and environmental exposures was available on 669 participants for FEV1 decline, and on 650 for FEV1/FVC and FEF25–75 decline.

We used a slightly modified version of the Adaptive Rank Truncation Product (ARTP) method described by Yu and colleagues [52] to calculate gene- and pathway level p-values. Briefly, according to the method, SNPs are sorted in ascending order of interaction strength, and SNP-interaction p-values are multiplied up to several pre-specified truncation points which depend on the number of SNPs in the gene. The statistical significance of these products is derived using the empirical distribution of products observed in the original and permutated datasets. For each gene, the strongest product p-value across all truncation points is readjusted using again its empirical distribution, to result in the gene-level p-value. Using the gene-level p-values in observed and permutated datasets, the same procedure can be applied to calculate pathway-level p-values. Details on the ARTP method, the applied modifications and truncation point definitions are presented in Figure S2 and Methods S1.

SNP-level analyses were run 10000 times, always after having newly permuted gene-specific SNP-allele-dosages across participants. SNP-level interaction p-values of the observed and permutated datasets were used for calculating gene- and pathway-level p-values. According to Yu et al. [52], results from simulation studies suggest the ARTP-method yields type I error rates close to 5%. We thus additionally corrected for 152 tests at the gene and 14 tests at the pathway level in a first look. In a second line of investigation, a non-stringent nominal threshold of α = 0.05 was chosen for further exploring gene- and pathway-level interaction signals due to our restricted sample size.

#### Comparing the impact of PM10 versus tobacco smoking

Emerging patterns of interaction were compared between exposures at the pathway- and gene-level. In pathways with nominally significant interactions, gene-level p-values were plotted against each other to identify the relative contributions to the pathway signal.

For the SNP with the strongest interaction signal in each nominally significant gene regression analyses were repeated with exposure centered to the median. Effect estimates were scaled to represent an exposure contrast of one interquartile range (IQR), and interaction effect sizes were compared between PM10 and tobacco smoke exposure. For SNP rs2035268 in gene SNCA, which was one of the top interaction signals in FEV1/FVC decline, genotype specific estimates for PM10 and packyears exposure were calculated to exemplify the effect modification by genotype. To this purpose, imputed allele dosages were coded as genotypes as follows: dosage <0.5 genotype TT, 0.5≤ dosage <1.5 genotype GT, and dosage ≥1.5 genotype GG. Reparametrization of exposure variables into genotype specific ones was employed to avoid model-overspecification and instable estimation in small genotype strata (rs2035268: MAF 5%).

#### Statistical power

Power calculations were done using QUANTO software [53] version 1.2 specifying a gene-environment study on independent individuals. Details of the power calculation are given in Methods S1. The most important aspect of the calculation was that a two-sided significance threshold of 5% was used (i.e. no multiple testing correction was included), since all 12679 SNP-estimates were further processed for deriving gene- and pathway level p-values without filtering by association strength. In our first analysis with 650 subjects, we have at least 75% power to detect a SNP*environment interaction that accounts for 1% of the total variance and that power increases to 99% when the SNP*environment interaction accounts for 5% of the total variance. In the replication analysis with n = 3320, estimated power is 99% in both cases. Statistical power is expected to be higher for the gene and pathway level analysis, but that increase in power could not be quantified since p-values for interaction at the gene (or pathway) level are obtained from individual p-values for interactions with SNPs belonging to the gene (or pathway), and the effect of interaction may vary among SNPs.

## Results

### Characteristics of Study Population

Regarding the distribution of sex, age and lung function according to categories of smoking and PM10 exposure, our study sample on average presented decreasing lung function values and accelerated lung function decline with increased smoking (**Table 2**). The percentage of females decreased with smoking exposure. Compared to participants assessed only at baseline, our study sample had slightly better lung function values, substantially less current smokers, was slightly less exposed to PM10 and tobacco smoke, and was older and leaner (**see Table S2**).

### SNP-level Analysis

A SNP-level analysis correcting for 12679 tests (α = 0.05/12679 = 3.9×10^−6^) detected an interaction between SNP rs2035268 in gene ***synuclein alpha*** (***SNCA***) on chromosome 4 q21 and PM10 on FEV_1_/FVC decline (p_interaction_ = 2.5×10^−6^). Compared to the baseline TT genotype, each G-allele was associated with a 3.8% (95% confidence interval (95%-CI) 2.2 to 5.4%) higher decline per 83.4 µg/m3*year PM10 exposure (IQR) over 11 years. Further, rs12190800 located in gene Parkinson disease protein 2 (*PARK2)* on chromosome 6 q25.2 interacted with PM10 on FEV_1_ decline. Compared to the TT-genotype each C-allele entailed an attenuation of 95.1 ml (95%-CI 60.1 ml to 130.1 ml) in FEV_1_ decline per IQR of PM10 (p_interaction_ = 9.8×10^−8^). Exposure and outcome specific regression estimates for all 12679 SNPs are given in **Data S1**.

### Gene-level Analysis

In the gene-level analysis, nominally interacting genes differed between PM10 and packyears exposure across the parameters of lung function decline (**Table 3**). Genes interacting with PM10 exposure partially overlapped for FEV_1_/FVC and FEF_25–75_ decline (genes *CRISP2*, *ERCC1*, *LPO*, *MPO*, and *SNCA*). After correcting for performing 152 gene-level tests (α_Bonferroni_ = 0.05/152 = 3.29*10^−4^), the interaction between gene ***cysteine-rich secretory protein 2*** (***CRISP2***) located on chromosome 6p12.3 and interval PM10 exposure on FEV_1_/FVC decline remained significant (p_interaction_ = 3.0×10^−4^). A marginally significant interaction was seen for gene *SNCA* on chromosome 4q21 with the same outcome and exposure (p_interaction_ = 4.0×10^−4^). Interactions observed for packyears exposure did not withstand multiple testing corrections.

**Table 3:** Nominally significant gene-environment interactions by outcome and exposure.

Outcome	decline FEV_1_ (n = 669)		decline FEV_1_/FVC (n = 650)		decline FEF_25–75_ (n = 650)	
Exposure	interval PM10	packyears	interval PM10	packyears	interval PM10	packyears
**Gene (p**_interaction_)	CP (0.005)	BCL2 (0.003)	**CRISP2 (0.0003)** a	PSMB5 (0.003)	LPO (0.008)	TGFBR2 (0.006)
	PRDX3 (0.010)	PTK2B (0.017)	**SNCA (0.0004)** b	SOD2 (0.015)	ERCC1 (0.014)	PTK2B (0.033)
	ERCC1 (0.014)	PSEN1 (0.023)	ERCC1 (0.007)	MAP2K1 (0.019)	MPO (0.018)	TP53 (0.033)
	RAC1 (0.027)	NOXO1 (0.034)	ALOX12 (0.012)	NFKB1 (0.022)	SLC23A2 (0.022)	CASP6 (0.039)
	CYP1A2 (0.028)	AOX1 (0.044)	LPO (0.018)	HMOX2 (0.048)	CRISP2 (0.023)	OXR1 (0.045)
	PSMB5 (0.038)	MAP2K1 (0.046)	CHUK (0.035)		SNCA (0.025)	TXNRD2 (0.047)
	GLRX (0.046)		GPX5 (0.039)		GPX5 (0.026)	
	GLRX2 (0.048)		MPO (0.039)		COL1A1 (0.049)	
			EPX (0.040)			

Genes are sorted in ascending order of interaction p-value within outcome-exposure strata.

a significant after Bonferroni-correction for testing 152 genes (α = .00033).

b marginally significant after Bonferroni-correction for testing 152 genes (α = .00033).

P-values of interaction for all tested genes are given in **Table S3**.

### Pathway-level Analysis

**Figure 1:**
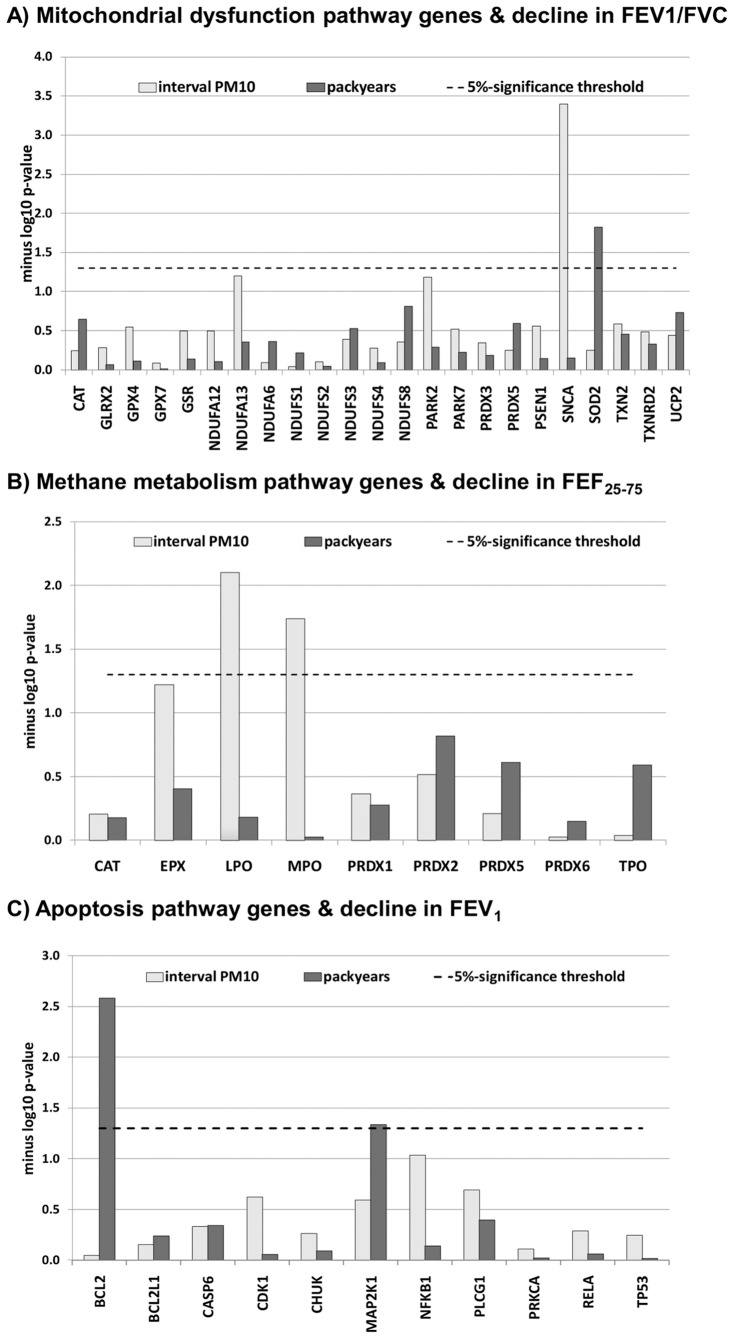
Distribution of interaction p-values across genes mapping to pathways with weak interaction signals. P-values of interaction on the gene-level are given on a minus log_10_ scale (y-axis), i.e. higher bars represent smaller interaction p-values. (A) Genes of the mitochondrial dysfunction pathway interacting with PM10 and packyears exposure between surveys on FEV_1_/FVC decline. (B) Genes of the methane metabolism pathway interacting with PM10 and packyears exposure between surveys on FEF_25–75_ decline. (C) Genes of the apoptosis signaling pathway interacting with PM10 and packyears exposure between surveys on FEV_1_ decline.

Pathways **“mitochondrial dysfunction”** and **“methane metabolism”** interacted nominally (α = 0.05) with PM10 on FEV_1_/FVC decline (p = 0.017) and FEF_25–75_ decline (p = 0.029), respectively. A further interaction signal was observed for pathway **“apoptosis”** and packyears exposure on FEV_1_-decline (p = 0.051). Inspecting the interaction p-values of pathway-specific genes revealed that the pathway signals mostly arose from single genes (*SNCA* in pathway “mitochondrial dysfunction”) or single genomic loci (overlapping gene regions of genes *eosinophil peroxidase, EPX, lactoperoxidase*, *LPO*, and *myeloperoxidase, MPO* in pathway “methane metabolism”) (**Figure 1** **, parts A–C**). P-values of interaction for all tested pathways are given in **Table S3**.

### Comparison of Interactions with PM10 Versus Packyears Exposure

The comparison of interaction effect sizes for PM10 and packyears exposure was based on regression estimates for the strongest interacting SNP only within each nominally significant gene. **Table 4** presents estimates for FEV_1_/FVC decline, where significant and marginally significant gene-level interactions have been detected for genes *CRISP2* and *SNCA*, respectively. Estimates for decline in FEV_1_ and FEF_25–75_ are presented in **Table S4 and Table S5**.

**Table 4:** Effect estimates of the strongest interacting SNP from each nominally significant gene on FEV_1_/FVC decline (n = 650).

Exposure	Chrom	Position	Gene	SNP	type	All1	All2	Freq All1	Beta_interaction_ (SE), P	Beta_SNP_ (SE), P	Beta_exposure_ (SE), P
PM10	4	90975104	***SNCA***	rs2035268	imp	G	T	0.05	**−3.8** (0.8), *2.54E-06* *	**−**0.7 (0.6), *0.254*	**−**0.2 (0.7), *0.786*
(IQR. 83.4	6	49766228	***CRISP2***	rs360563	imp	C	T	0.50	**−1.1** (0.3), *3.78E-05*	0.0 (0.3), *0.975*	0.6 (0.7), *0.375*
ug/m3 * y)	17	6840800	***ALOX12***	rs2073438	gen	A	G	0.26	**1.0** (0.3), *2.38E-04*	0.4 (0.3), *0.181*	**−**1.0 (0.7), *0.144*
	17	53675156	***LPO***	rs8178290	imp	A	C	0.18	**1.1** (0.3), *9.61E-04*	**−**0.2 (0.3), *0.582*	**−**1.0 (0.7), *0.153*
	17	53629132	***EPX***	rs3785496	gen	A	G	0.80	**−1.1** (0.3), *0.001*	**−**0.1 (0.3), *0.773*	1.1 (0.8), *0.140*
	17	53699864	***MPO***	rs8178409	imp	A	G	0.18	**1.1** (0.3), *0.001*	**−**0.2 (0.3), *0.523*	**−**1.0 (0.7), *0.158*
	19	50600888	***ERCC1***	rs1005165	imp	C	T	0.83	**−1.3** (0.4), *0.002*	0.1 (0.4), *0.765*	1.5 (0.9), *0.084*
	6	28629296	***GPX5***	rs393414	gen	C	T	0.79	**−0.9** (0.3), *0.003*	0.3 (0.3), *0.385*	1.0 (0.8), *0.219*
	10	101996416	***CHUK***	rs4919438	imp	C	T	0.50	**−0.8** (0.3), *0.003*	**−**0.1 (0.2), *0.669*	0.2 (0.7), *0.813*
packyears	14	22552780	***PSMB5***	rs12590429	imp	A	G	0.09	**−3.8** (0.9), *1.06E-05*	0.3 (0.5), *0.540*	**−**0.5 (0.5), *0.265*
(IQR: 9.8	6	160020288	***SOD2***	rs7855	imp	A	G	0.94	**2.7** (0.7), *2.17E-04*	**−**0.4 (0.8), *0.620*	**−**6.1 (1.4), *1.64E-05*
PY)	15	64583032	***MAP2K1***	rs8043062	imp	A	G	0.15	**1.9** (0.6), *0.001*	**−**0.1 (0.3), *0.741*	**−**1.6 (0.5), *0.003*
	4	103676616	***NFKB1***	rs230528	gen	G	T	0.38	**−1.7** (0.6), *0.003*	0.1 (0.3), *0.775*	0.2 (0.6), *0.693*
	16	4466293	***HMOX2***	rs2270363	imp	A	G	0.25	**1.1** (0.4), *0.013*	0.0 (0.3), *0.935*	**−**1.8 (0.6), *0.002*

SNP-estimates are based on an additive model. Beta-estimates represent percentages of decline in FEV_1_/FVC over 11 years per effect allele and/or for an exposure contrast of one interquartile range (IQR). All estimates are taken from the same interaction model. Positive values mean an attenuation, and negative ones an acceleration of FEV_1_/FVC decline. Rows are sorted according to ascending interaction p-values.

* significant after Bonferroni correction for testing 12679 SNPs (α = 3.9×10E-6).

gen: genotyped SNP; imp: imputed SNP; All1: allele 1 (effect allele); All2: allele 2 (baseline allele); FreqAll1: frequency of allele 1.

The C-allele of SNP rs360563 in gene *CRISP2* accelerated FEV_1_/FVC decline by 1.1% per IQR change in PM10 exposure over 11 years (**Table 4**). Similarly, the G-allele of SNP rs2035268 in *SNCA* was associated with an accelerated decline by 3,8% per allele and IQR change in exposure. Genotype specific exposure estimates were calculated for rs2035268. Within genotypes GT and GG of SNP rs2035268, a change in IQR of PM10 was associated with a signficant acceleration of FEV_1_/FVC decline by 3.9%, opposed to a small and non-signficiant acceleration by 0.2% in baseline genotype TT (**Table 5**). In contrast, a change in IQR of packyears smoked was associated with a significant acceleration by 1.1% in the baseline TT genotype stratum, but not in the GT/GG strata.

**Table 5:** rs2035268 genotype specific estimates of the effect of interval PM10 and pack years exposure on percentage decline in FEV_1_/FVC ratio during 11 years of follow-up.

exposure	rs2035268 genotype	effect estimate^a^	(95%-confidence interval)	p-value	p_interaction_ b
**interval PM10**	wild-type (TT)	**−0.2**	(−1.7 to 1.4)	0.827	**7.35E-07**
(IQR 83.4ug/m^3^* y)	mutant (GT/GG)	**−3.9**	(−5.9 to −1.8)	2.25E-04	
**packyears**	wild-type (TT)	**−1.1**	(−2.0 to −0.1)	0.024	0.909
(IQR 9,8 PY)	mutant (GT/GG)	**−0.7**	(−2.3 to 1.0)	0.434	

a Environmental effect estimates are based on a multiple linear model with sample size n = 650 adjusting for sex, age and height at follow-up, packyears smoked up to baseline, population ancestry, and study area. PM10 and packyears exposure was reparametrized into genotype specific exposure variables to avoid model overspecification with instable estimates in the genotypic risk stratum (rs2035268 has minor allele frequency of 0.05). Estimates are in units of percentage decline in FEV_1_/FVC.

b p-value of interaction between environmental exposure and genotypes of rs2035268 (TT vs GT/GG).

For FEV_1_- and FEF_25–75_ decline, interaction effect sizes for the strongest interacting SNPs in nominally significant genes tended to be considerably larger with packyears compared to PM10 exposure. Further, packyears exposure frequently presented significant main effects besides the interaction with SNPs (**Table S4 and Table S5**).

In models including only main effects but no interaction between SNPs and exposure, an IQR of 9.8 packyears was significantly associated with accelerated decline in FEV_1_/FVC by 1%, and in FEV_1_ by 50 ml (data not shown). Respective estimates for PM10 were non-significant. SNP main effects remained non-significant and their beta estimates largely unaffected by the exclusion of interaction terms.

### Replication of Significant Associations

Replication genotyping was done for *CRISP2* SNP rs360563 (MAF of 49.8%) and rs12190800 in *PARK2* (MAF 16%), but their interaction with PM10 exposure on FEV_1_/FVC and FEV_1_ decline could not be confirmed in the remainder of the SAPALDIA population (p_interaction_ = 0.63 and 0.50 respectively, n = 3320 for both). Thereby, MAFs in the replication sample corresponded to those in the discovery sample, and both SNPs were in Hardy-Weinberg equilibrium.

## Discussion

To the best of our knowledge, this is the first study assessing gene-environment interactions on lung function decline using analysis methods that accumulate interaction effects along a broadly defined set of candidate genes and pathways. Our results suggest that different oxidative stress genes could be involved in mediating the adverse effects of ambient air pollution and tobacco smoke exposure on lung function decline.

We can currently only hypothesize about the reason for observing different patterns of interaction between the two environmental exposures. A possible explanation would be that ambient particulate matter pollution and tobacco smoke, although sharing many constituents, also differ in their composition, which possibly affects the overall and relative relevance of the different pathways. A probably more important explanation is that levels of oxidative stress imposed by ambient PM10 exposure are much lower than those induced by active tobacco smoking. Experimental studies have shown that different levels of oxidative stress trigger dose-dependent, specific activations of pathways on the cellular level in response to the oxidant burden [54]. Li and colleagues delineated a **stratified oxidative stress** **model** while studying the biological effects of particulate matter exposure on human and mouse cell lines exposed to solutions of Diesel exhaust particles (DEP) and concentrated ambient air particles (CAP) sampled in a highly polluted area [55], [56]. According to their observations, at the lower end of exposure pivotal ROS-scavenging enzymes like heme oxygenase-1 are induced, representing the activation of protective cell-mechanisms. Intermediate exposure levels trigger inflammatory pathways via signal transduction cascades (increased expression of interleukin-8 and Jun kinase), while high exposure levels impact on mitochondrial permeability, and result in cytotoxicity and apoptosis. Thereby CAP were mostly representing the lower to mid-level of exposure, inducing oxidative-stress enzymes and inflammation, but not apoptosis (as observed with DEP). In contrast, tobacco smoke exposure is known to induce the whole spectrum of cellular reactions, from oxidative stress response and inflammation [57], [58] up to DNA-damage [58], apoptosis [59], [60], [61], [62] as well as cellular necrosis [61]. Although in the light of limited sample size, we cannot provide statistical evidence of exposure-specific interaction patterns with genes and pathways in our current study, it is interesting to see that many of the top-ranking genes interacting with packyears exposure are involved in signal transduction or apoptosis (**Table 3** *BCL2, CASP6, MAP2K1, NFκB1, TGFBR2, TP53*). Only two such genes showed interaction signals with PM10 exposure (*CHUK, RAC1*), and many of the others related to scavenging or production of ROS (*CRISP2, CYP1A2, EPX, GLRX, GLRX2, GPX5, LPO, MPO, PRDX3*). These observations are consistent with the stratified oxidative stress model. The observation of larger interaction effect sizes at the level of SNPs for FEV_1_ and FEF_25–75_ decline, as well as the frequent presence of significant main effects further support higher oxidative stress levels induced by tobacco smoke than PM10 exposure.

Another important observation was that the effect of genetic variation related to oxidative stress appeared to be mediated predominantly by the interactions with environmental exposures, as hardly any SNP main-effects were observed. This is in line with the findings of genome-wide studies on lung function performed to date [17], [18], [21], where oxidative-stress related candidate genes did not produce strong signals. But their design was cross-sectional and importantly, these analyses focused on SNP-main effects. Exposure specific gene-effects might thus be missed as they can cancel out when averaged over the whole population (which happens in a gene main effect analysis). Disregarding gene-environment interaction might also explain part of the missing heritability in complex disease genetics.

Our study had several limitations. First, the limited number of non-asthmatic adults with available genome-wide data restricted our power to detect associations at the gene and pathway levels. In this context, we faced the problem of finding studies with genome-wide genotyping and comparable data on both phenotypes and environmental exposures. This issue is particularly imminent regarding ambient air pollution exposure. As a consequence, small sample size did not allow us to identify further strong interaction signals to follow-up, while the observed ones could not be replicated in the remainder of the study population. Our gene and pathway level results are thus of more exploratory nature. Limited power is also known to inflate effect estimates when the strongest association signals are selected for further follow-up (so-called “winner’s curse” [63]), thus our interaction effect estimates on the SNP-level are likely overestimated for both exposures. But the relative difference in effect size between exposures is probably less affected by this phenomenon. In case of differential overestimation, the true difference would likely be larger, as observed PM10 effects were smaller and therefore would be more affected than packyears effects. Further, follow-up participants were healthier than those completing only baseline examination. Our results are thus applicable to an adult general population sample of good health. Environmental exposure and genetic susceptibility might possibly have affected health and thus participation of our study subjects. But in this case, true effects would likely be underestimated in our present study [64]. Finally, SNP-coverage was low for certain genes (**see Table S1**), and the well-known gene-deletions in glutathione S-transferases are difficult to tag by SNP-genotyping as they represent copy number variations. This makes it difficult to interpret respective results. On the other side, a comparison of imputed SNP data for rs360563 (gene *CRISP2*) with genotypes measured during replication in the initial study sample showed a high concordance indicating high imputation quality (**see Table S6**). The absence of strong interactions on the pathway level is likely due to our primary focus on function while selecting candidate genes, which limited pathway coverage. But genes in a pathway may also differently interact with exposure, or compensate for each other. Further, regulatory genomic regions could be located farer away than the chosen flanking segments of 20 kilobases. Detecting interactions in pathways is thus more challenging.

Strengths of our study were the population based design comprising non-asthmatic adults of a wide age-spectrum, the detailed data on individual tobacco smoke and particularly PM10 exposure, and the high quality of longitudinal lung function data. Finally, the application of analysis methods which exploit interaction signals below the significance threshold of a pure SNP-level analysis provided new insight into a possible differential involvement of genes according to exposure specific oxidative stress levels.

### Conclusions

Applying a gene- and pathway-level analysis, we observed that PM10 and packyears exposure potentially interact with different genes on lung function decline, consistent with a stratified response to different oxidative stress levels. Our study thus points to the importance of considering interactions with environmental factors in the search for molecular pathways underlying lung function decline in response to exogenous inhalants. But it is also a good example of the challenges faced by gene-environment interaction studies today: While studies with partial genome-wide data, and hence often small sample size, can beneficially use the remainder of the study population as highly comparable replication sample, their potential to identify sufficient variants to follow-up is limited. In contrast, large studies or study consortia are more powerful in the discovery stage, but suffer from data heterogeneity as finding suitable replication studies with comparable phenotypic, genetic and environmental exposure data is difficult. This results in a challenging trade-off between sample size and data homogeneity.

## Supporting Information

Figure S1 **Follow-up of participants and selection of study population.**
(TIF)

Figure S2 **Scheme of analysis steps in the ARTP-method.** The ARTP method developed by Yu and colleagues [52] assumes that an analysis at the SNP-level has been performed on the originally observed data, followed by a reanalysis on permutated datasets, i.e. p-values of association for original and permutated datasets are available for each SNP. The ARTP procedure then entails the following 4 steps: 1. **Order** p-values from single SNP analysis in ascending order, 2. Calculate **products** of ranked p-values at different truncation points depending on gene length (light and dark green arrows in the graph), 3. **Adjust** product p-values using permutation distribution (1^st^ and 2^nd^ yellow arrow from the right), 4. **Select** the **minimum** of the adjusted products (red arrow) and readjust (1^st^ yellow arrow from the left). The **readjusted product minimum** represents the **gene-level p-value**. For each permutated dataset, an adjusted product minimum can be calculated as well. The procedure can then be repeated using the resulting, original and permutation gene-level p-values to yield p-values of the pathway.
(TIF)

Table S1 Characteristics of selected oxidative-stress related genes and mapping to candidate pathways.
(XLS)

Table S2 Comparison of study sample to non-asthmatic participants lost to follow-up, and those followed-up w/o genome-wide data. P_A-C_ p-value for comparisons of characteristics between baseline and analysis sample; P_B-C_ p-value for comparisons of characteristics between follow-up and analysis sample; ^a^ Chi-squared tests for proportions, two sample T-tests for means, and ranksum-test for medians, ^b^ n = 650 with complete baseline and follow-up data, **^c^** in ever-smokers only.
(XLS)

Table S3 Interaction P-values by lung function decline parameter and exposure for a) all tested pathways and b) genes.
(XLS)

Table S4 Effect estimates of the strongest interacting SNP from each nominally significant gene on FEV_1_-decline (n = 669). The table shows the effect estimates of the strongest interacting SNP in each nominally significant gene (i.e. with a gene p-value for interaction <0.05). SNP-estimates are based on an additive model. Beta-estimates are in units of milliliters for FEV_1_, and represent declines per effect allele and/or for an exposure contrast of one interquartile range (IQR) over 11 years. All estimates are taken from the same interaction model. Positive values mean that the respective decline is attenuated, opposed to acceleration with negative values. Rows are sorted according to ascending interaction p-values. gen: genotyped SNP; imp: imputed SNP; All1: allele 1 (effect allele), All2: allele 2 (baseline allele); FreqAll1: frequency of allele 1.
(DOC)

Table S5 Effect estimates of the strongest interacting SNP from each nominally significant gene on FEF_25–75_-decline (n = 650). The table shows the effect estimates of the strongest interacting SNP in each nominally significant gene (i.e. with a gene p-value for interaction <0.05). SNP-estimates are based on an additive model. Beta-estimates are in units of milliliters per second, and represent declines per effect allele and/or for an exposure contrast of one interquartile range (IQR) over 11 years. Positive values mean that the respective decline is attenuated, opposed to an acceleration with negative values. gen: genotyped SNP; imp: imputed SNP; All1: allele 1 (effect allele), All2: allele 2 (baseline allele); FreqAll1: frequency of allele 1.
(DOC)

Table S6 Comparison of genotyped and imputed data for SNP rs360563 in gene CRISP2. *imputed allele dosages have been rounded to corresponding integer values of mutant alleles as follows:
dosage <0.500
> = 0.5 and <1.5
1
> = 1.52N = 665 corresponds to the current analysis sample with available genotyping data.
(DOC)

Methods S1 Details on the ARTP method specifications and power calculations.
(DOC)

Text S1 Overview of the SAPALDIA study team as of July 2011.
(DOC)

Data S1 Outcome and exposure specific regression results of all 12679 SNPs. Effect estimates are derived from multiple linear regression models specifying SNP main effects, interval PM10 and packyears exposure (centered to the median) and an interaction between SNP and either PM10 or packyears exposure. An additive genetic model was assumed. Adjustments were made for sex, age and height at follow-up, packyears smoked up to baseline, population ancestry, and study area. Beta-estimates are in units of milliliters for FEV_1_, percentages for FEV_1_/FVC, and milliliters per second for FEF_25–75_, Betas represent declines per effect allele and/or for an exposure contrast of one interquartile range (IQR) over 11 years. All estimates are taken from the same interaction model. Positive values mean that the respective decline is attenuated, opposed to acceleration with negative values. Rows are sorted according to chromosome and position. All1: allele 1 (effect allele), All2: allele 2 (baseline allele); FreqAll1: frequency of allele 1, MAF: minor allele frequency. n: number of observations in the model. Beta_int/se_int/p_int: beta estimate/standard error/p-value of the SNP*environment interaction term.
(XLS)
