# Supplementary figures and images for: Different Genes Interact with Particulate Matter and Tobacco Smoke Exposure in Affecting Lung Function Decline in the General Population
Source: PLoS One. 2012 Jul 6;7(7):e40175. doi: 10.1371/journal.pone.0040175 (PMC3391223; doi:10.1371/journal.pone.0040175)

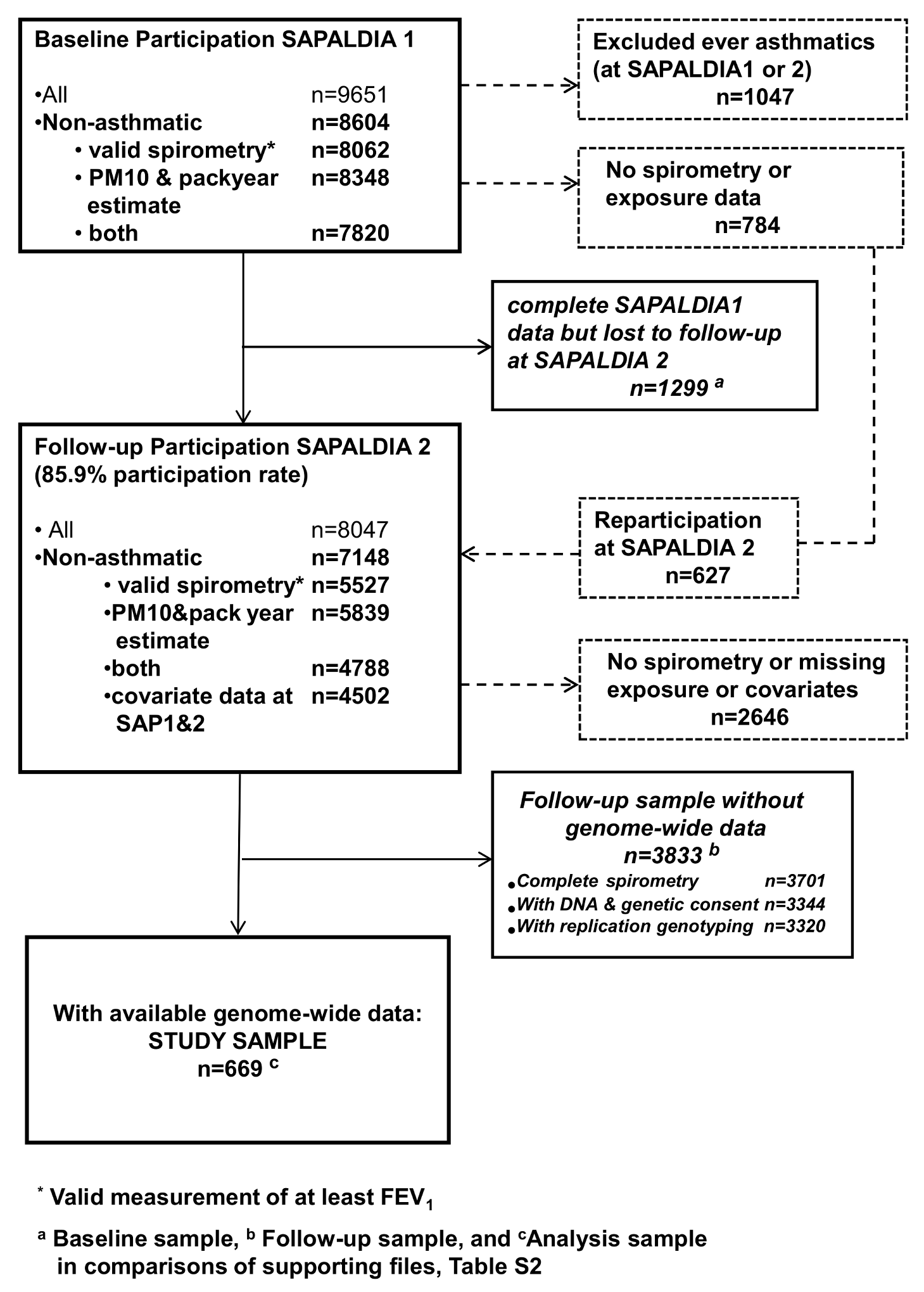

Supplement: Figure S1 — Follow-up of participants and selection of study population. (TIF) [file pone.0040175.s001.tif]

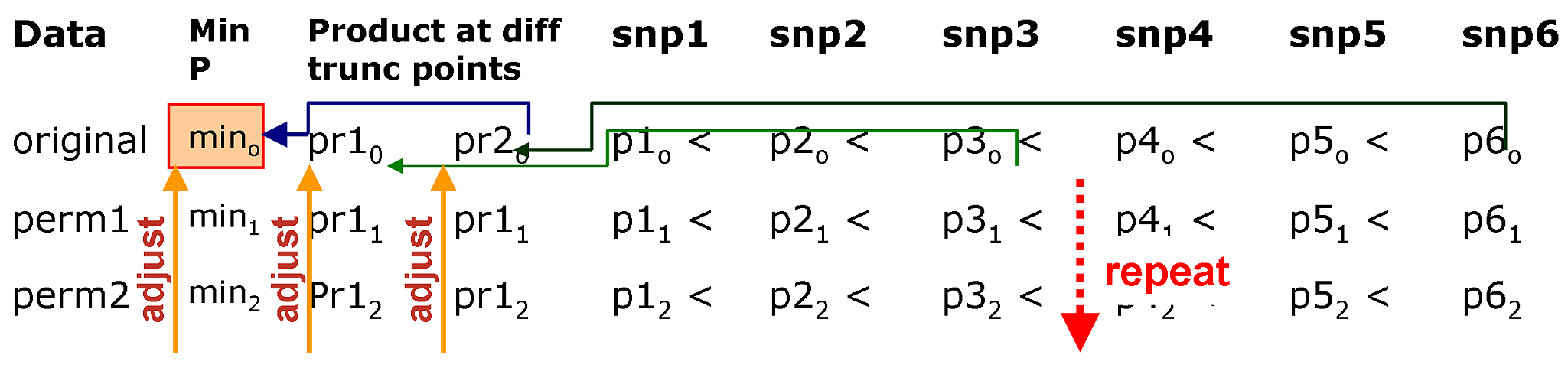

Supplement: Figure S2 — Scheme of analysis steps in the ARTP-method. The ARTP method developed by Yu and colleagues [52] assumes that an analysis at the SNP-level has been performed on the originally observed data, followed by a reanalysis on permutated datasets, i.e. p-values of association for original and permutated datasets are available for each SNP. The ARTP procedure then entails the following 4 steps: 1. Order p-values from single SNP analysis in ascending order, 2. Calculate products of ranked p-values at different truncation points depending on gene length (light and dark green arrows in the graph), 3. Adjust product p-values using permutation distribution (1st and 2nd yellow arrow from the right), 4. Select the minimum of the adjusted products (red arrow) and readjust (1st yellow arrow from the left). The readjusted product minimum represents the gene-level p-value. For each permutated dataset, an adjusted product minimum can be calculated as well. The procedure can then be repeated using the resulting, original and permutation gene-level p-values to yield p-values of the pathway. (TIF) [file pone.0040175.s002.tif]
