# Supplementary material for: Different Genes Interact with Particulate Matter and Tobacco Smoke Exposure in Affecting Lung Function Decline in the General Population
Source: PLoS One. 2012 Jul 6;7(7):e40175. doi: 10.1371/journal.pone.0040175 (PMC3391223; doi:10.1371/journal.pone.0040175)
Supplement: Table S4 — Effect estimates of the strongest interacting SNP from each nominally significant gene on FEV1-decline (n = 669). The table shows the effect estimates of the strongest interacting SNP in each nominally significant gene (i.e. with a gene p-value for interaction <0.05). SNP-estimates are based on an additive model. Beta-estimates are in units of milliliters for FEV1, and represent declines per effect allele and/or for an exposure contrast of one interquartile range (IQR) over 11 years. All estimates are taken from the same interaction model. Positive values mean that the respective decline is attenuated, opposed to acceleration with negative values. Rows are sorted according to ascending interaction p-values. gen: genotyped SNP; imp: imputed SNP; All1: allele 1 (effect allele), All2: allele 2 (baseline allele); FreqAll1: frequency of allele 1. (DOC) [file pone.0040175.s006.doc]

**Table S4 Effect estimates of strongest interacting SNPs from nominally significant genes regarding FEV_1_ decline (n=669).**

| **Exposure** | **Chrom** | **Position** | **gene** | **SNP** | **type** | **All1** | **All2** | **Freq All1** | **Beta_interaction_, (SE), P** | **Beta_SNP_ , (SE), P** | **Beta_exposure_, (SE), P** |
| --- | --- | --- | --- | --- | --- | --- | --- | --- | --- | --- | --- |
| Interval | 3 | 150398272 | **CP** | rs13075891 | imp | A | T | 0.11 | **-96.**3 (23.6), 4.65E-05 | 46.3 (23.4), 0.048 | 93.3 (53.0), 0.078 |
| PM10 | 10 | 120928816 | **PRDX3** | rs1553850 | imp | A | T | 0.59 | **48.9** (14.7), 8.38E-04 | -23.8(15.9), 0.136 | 20.3 (55.4), 0.713 |
| (IQR: 83.4 | 19 | 50625332 | **ERCC1** | rs11882642 | gen | C | T | 0.39 | **50.7** (16.4), 0.002 | -6.1 (14.6), 0.676 | 30.5 (55.9),0.586 |
| ug/m^3^* y) | 5 | 95176000 | **GLRX** | rs1047420 | imp | A | G | 0.39 | **-45.5** (15.3), 0.003 | 32.8 (15.0), 0.029 | 107.5 (54.2), 0.047 |
|  | 7 | 6373458 | **RAC1** | rs6463553 | imp | A | G | 0.14 | **71.8** (24.9), 0.004 | 13.5 (21.4), 0.529 | 55.3 (53.8), 0.304 |
|  | 15 | 72831288 | **CYP1A2** | rs2472304 | gen | A | G | 0.63 | **41.1** (14.5), 0.004 | -16.8 (15.5), 0.277 | 24.4 (56.4), 0.665 |
|  | 14 | 22564116 | **PSMB5** | rs8013143 | gen | A | G | 0.72 | **-43.7** (15.8), 0.006 | 11.5 (16.2), 0.478 | 132.7 (57.1), 0.020 |
|  | 1 | 191350672 | **GLRX2** | rs10733078 | imp | C | T | 0.68 | **41.0** (16.4), 0.013 | -9.5(16.1), 0.557 | 19.1 (57.1), 0.738 |
| Packyears | 14 | 72773504 | **PSEN1** | rs177408 | imp | C | G | 0.38 | **124.4** (31.9), 9.78E-05 | 0.5 (15.7), 0.973 | -150.9 (39.5),1.32E-04 |
| (IQR: 9.8 | 18 | 58953912 | **BCL2** | rs4941183 | gen | A | G | 0.53 | **-104.7** (29.6), 4.09E-04 | 6.1 (14.6), 0.678 | 64.1 (44.1), 0.146 |
| PY) | 8 | 27337100 | **PTK2B** | rs4434577 | imp | C | T | 0.83 | **144.3** (44.2), 0.001 | -3.3(18.8), 0.862 | -291.5 (77.4), 1.67E-04 |
|  | 2 | 201194080 | **AOX1** | rs6761375 | gen | A | G | 0.10 | **-208.7** (70.4), 0.003 | -9.1(28.9), 0.754 | -23.6 (30.7), 0.441 |
|  | 16 | 1961894 | **NOXO1** | rs2302178 | gen | A | G | 0.85 | **142.1** (47.2), 0.003 | -7.9(20.3), 0.696 | -289.0 (88.6), 0.001 |
|  | 15 | 64483384 | **MAP2K1** | rs11856510 | imp | G | T | 0.08 | **106.7** (39.3), 0.007 | 3.0 (24.0), 0.900 | -66.6 (32.2), 0.039 |
|  |  |  |  |  |  |  |  |  |  |  |  |
