# Supplementary material for: Different Genes Interact with Particulate Matter and Tobacco Smoke Exposure in Affecting Lung Function Decline in the General Population
Source: PLoS One. 2012 Jul 6;7(7):e40175. doi: 10.1371/journal.pone.0040175 (PMC3391223; doi:10.1371/journal.pone.0040175)
Supplement: Table S5 — Effect estimates of the strongest interacting SNP from each nominally significant gene on FEF25–75-decline (n = 650). The table shows the effect estimates of the strongest interacting SNP in each nominally significant gene (i.e. with a gene p-value for interaction <0.05). SNP-estimates are based on an additive model. Beta-estimates are in units of milliliters per second, and represent declines per effect allele and/or for an exposure contrast of one interquartile range (IQR) over 11 years. Positive values mean that the respective decline is attenuated, opposed to an acceleration with negative values. gen: genotyped SNP; imp: imputed SNP; All1: allele 1 (effect allele), All2: allele 2 (baseline allele); FreqAll1: frequency of allele 1. (DOC) [file pone.0040175.s007.doc]

**Table S5 Effect estimates of strongest interacting SNPs from nominally significant genes regarding FEF_25-75_ decline.**

| **Exposure** | **Chrom** | **Position** | **gene** | **SNP** | **type** | **All1** | **All2** | **Freq**  **All1** | **n** | **Beta_interaction_, (SE), P** | **Beta_SNP_, (SE), P** | **Beta_exposure_, (SE), P** |
| --- | --- | --- | --- | --- | --- | --- | --- | --- | --- | --- | --- | --- |
| PM10 | 17 | 53678676 | **LPO** | rs8178307 | imp | C | T | 0.82 | 650 | **-160.0** (44.4), 3.13E-04 | 34.5 (48.5), 0.477 | 196.2 (112.9), 0.082 |
| (IQR: 83.4 | 20 | 4835900 | **SLC23A2** | rs1715386 | imp | C | T | 0.05 | 650 | **164.2** (45.8), 3.39E-04 | 25.0 (44.0), 0.570 | -305.6 (121.9), 0.012 |
| ug/m^3^* y) | 17 | 53699864 | **MPO** | rs8178409 | imp | A | G | 0.18 | 650 | **160.5** (45.8), 4.54E-04 | -36.5 (49.2), 0.458 | -124.2 (103.2), 0.229 |
|  | 4 | 90932280 | **SNCA** | rs17016168 | imp | A | G | 0.05 | 650 | **-321.0** (97.7), 0.001 | -96.1 (86.7), 0.268 | -36.2 (98.4), 0.713 |
|  | 6 | 28629296 | **GPX5** | rs393414 | gen | C | T | 0.79 | 650 | **-138.8** (42.5), 0.001 | 54.4 (43.7), 0.212 | 164.2 (118.9), 0.167 |
|  | 17 | 45617120 | **COL1A1** | rs1061947 | gen | A | G | 0.16 | 650 | **143.2** (46.6), 0.002 | -29.5 (53.8), 0.584 | -106.3(100.9), 0.292 |
|  | 6 | 49771424 | **CRISP2** | rs2248263 | imp | A | G | 0.27 | 650 | **118.8** (39.9), 0.003 | 43.4 (43.4), 0.318 | -90.6 (100.1), 0.366 |
|  | 19 | 50616200 | **ERCC1** | rs3212948 | imp | C | G | 0.80 | 650 | **114.4** (40.1), 0.004 | -54.5 (37.2), 0.143 | -146.7 (101.8), 0.150 |
| Packyears | 3 | 30700648 | **TGFBR2** | rs876687 | gen | C | T | 0.11 | 650 | **-374.9** (89.9), 3.01E-05 | 55.4 (48.1), 0.250 | -60.4 (68.5), 0.378 |
| (IQR: | 8 | 107792912 | **OXR1** | rs776953 | imp | A | C | 0.80 | 650 | **272.3** (80.3), 6.99E-04 | 12.1 (46.0), 0.793 | -548.6 (152.7), 3.28E-04 |
| 9.8 PY) | 17 | 7500402 | **TP53** | rs1641511 | imp | A | G | 0.79 | 650 | **-249.6** (78.5), 0.001 | 39.8 (42.3), 0.347 | 294.7 (136.4), 0.031 |
|  | 8 | 27320584 | **PTK2B** | rs7006244 | imp | A | G | 0.93 | 650 | **444.8** (142.5), 0.002 | -154.3 (86.6), 0.075 | -944.3 (277.1), 6.55E-04 |
|  | 22 | 18300360 | **TXNRD2** | rs9606186 | imp | C | G | 0.45 | 650 | **-234.5** (77.3), 0.002 | -68.4 (38.3), 0.074 | 95.5 (86.6), 0.270 |
|  | 4 | 110835360 | **CASP6** | rs3181345 | imp | C | T | 0.91 | 650 | **387.6** (133.8), 0.004 | -41.6 (64.9), 0.521 | -812.1 (256.5), 0.002 |
