# Supplementary material for: Different Genes Interact with Particulate Matter and Tobacco Smoke Exposure in Affecting Lung Function Decline in the General Population
Source: PLoS One. 2012 Jul 6;7(7):e40175. doi: 10.1371/journal.pone.0040175 (PMC3391223; doi:10.1371/journal.pone.0040175)
Supplement: Table S6 — Comparison of genotyped and imputed data for SNP rs360563 in gene CRISP2. *imputed allele dosages have been rounded to corresponding integer values of mutant alleles as follows: dosage <0.500 > = 0.5 and <1.5 1 > = 1.52N = 665 corresponds to the current analysis sample with available genotyping data. (DOC) [file pone.0040175.s008.doc]

**Table S6 Comparison of genotyped and imputed data for SNP rs360563 in gene CRISP2.**

| **rs360563 genotypes** | **Rounded imputation allele dosages* [number** *(cell percentage)***]** | | |  | **Dosage distribution by genotype** | | |
| --- | --- | --- | --- | --- | --- | --- | --- |
|  | **0** | **1** | **2** | **Total** | **median** | **25. perc.** | **75. perc.** |
| TT | **164** *(24.7)* | 2 *(0.3)* | 0 *(0.0)* | 166 *(24.96)* | 0.017 | 0.006 | 0.026 |
| CT | 1 *(0.2)* | **318** *(47.8)* | 0 *(0.0)* | 319 *(48.0)* | 1.002 | 1.000 | 1.010 |
| CC | 0 *(0.0)* | 1 *(0.2)* | **179** *(26.9)* | 180 *(27.1)* | 1.998 | 1.998 | 1.999 |
| **Total** | 165 *(24.8)* | 321 *(48.3)* | 179 *(26.9)* | **665** *(100.0)* | 1.002 | 0.660 | 1.984 |
