# Supplementary material for: Different Genes Interact with Particulate Matter and Tobacco Smoke Exposure in Affecting Lung Function Decline in the General Population
Source: PLoS One. 2012 Jul 6;7(7):e40175. doi: 10.1371/journal.pone.0040175 (PMC3391223; doi:10.1371/journal.pone.0040175)
Supplement: Methods S1 — Details on the ARTP method specifications and power calculations. (DOC) [file pone.0040175.s009.doc]

**Adaptations to the ARTP-method**

We modified the method published by Yu slightly in our analysis: First, as our goal was to explore gene-environment interaction, our SNP-level association statistic was the p-value for interaction between SNP and environmental exposure (cumulative PM10 or tobacco smoke exposure). Second, instead of permuting the outcome variable, we permuted the genetic data. This allowed on the one side to properly adjust the outcome variables for known determinants, and on the other side to break up any correlations between SNPs and model covariates, which might bias the SNP-estimates. Permutation was done by randomly assigning the whole SNP-data of one individual to another one, thus keeping data coming from the same person together. In this way, linkage disequilibrium between gene-specific SNPs was preserved (as is the case when permuting outcomes).

Truncation points were chosen in a similar way as described by Yu et al. For pathways consisting of less than 5 genes, only the most strongly associated gene was considered, for those with <20 genes, p-value products were calculated on the first five. In case of 20 genes or more, the integer resulting from rounding off the ratio (number of pathway-genes)/20 was multiplied by 1, 2, …, 5 (e.g. for a pathway consisting of 40 genes, truncation points would be calculated at genes 2, 4, 6, 8, 10). Truncation points were defined similarly for SNPs: only the strongest SNP was considered if the gene had <10 SNPs available, between 10 and <20 SNPs, products were calculated on the strongest ten. In case of 20 SNPs or more, the number of SNPs was divided by 20 and the resulting integer multiplied by integers 1, 2, .., 9, 10, giving 10 truncation points.

SNP level analyses on decline in FEV1, FEV1/FVC and FEF_25-75_ were done using multiple linear regression in ProbABEL v0.1.3 including sex, age and height at follow-up, packyears smoked up to baseline and between surveys, PM10-exposure during follow-up, principal components of population ancestry, study areas, additive SNP main effect, and an interaction term between SNP and either PM10 or packyears exposure during follow-up. Robust sandwich-estimation of standard errors was specified in ProbABEL. SNP-level interaction p-values were then processed according to the ARTP method with 10’000 permutations to calculate gene and pathway p-values.

**Power calculations**

For the analyses on the SNP-level, power calculations were done using QUANTO software version 1.2. We specified a gene-environment study on independent individuals, and used the observed variability of outcome and environmental exposure in our data as input parameters. Since all 12679 SNP-estimates were further processed for calculating gene- and pathway level p-values without filtering by strength of association, our power calculations were based on single SNP tests. We thus assumed a two-sided significance threshold of 5% and did not apply multiple testing correction. An additive genetic model was used. Regarding our outcome model, we assumed that in a study of oxidative-stress genes, the marginal contribution of independent genetic effects (i.e. the part independent of gene-environment interaction) to explained total outcome variability would be negligible (0.05% was specified as marginal R-squared value), compared to the impact of environmental factors (5-15%), and gene-environment interaction (1-5%). Thereby the independent contribution of the environment impacted little on estimated power for detecting gene-environment interaction. In the first level analysis with N=650, power estimates were 75% and 99% for 1 and 5% of explained outcome variability by the SNP*environment interaction, respectively. The estimate was 99% in both cases for the replication analysis with N=3320.

Regarding gene and pathway level analysis, which constituted the main focus of our paper, we expected that the use of the ARTP method to derive interaction p-values on gene- and pathway levels results in a further increase in power, as SNP-level signals are accumulated onto higher analysis levels. In their original publication, the authors of the algorithm showed that partitioning the ARTP method into a gene and pathway level in most cases resulted in higher statistical power compared to an application on the SNP level only. However, it was not possible for us to quantify the exact increase in power resulting from use of the ARTP-method compared to a classic SNP-level analysis, as a) different units of analysis are concerned (SNPs versus genes and pathways) and b) the method only calculates overall p-values of interaction, but not effect sizes.

**References**
