# Supplementary material for: Different Genes Interact with Particulate Matter and Tobacco Smoke Exposure in Affecting Lung Function Decline in the General Population
Source: PLoS One. 2012 Jul 6;7(7):e40175. doi: 10.1371/journal.pone.0040175 (PMC3391223; doi:10.1371/journal.pone.0040175)
Supplement: Text S1 — Overview of the SAPALDIA study team as of July 2011. (DOC) [file pone.0040175.s010.doc]

## Current SAPALDIA Team

**Study directorate**: T Rochat (p), , JM Gaspoz (c), N Künzli (e/exp), NM Probst Hensch (e/g), C Schindler (s).

**Scientific team**: JC Barthélémy (c), W Berger (g), R Bettschart (p), A Bircher (a), G Bolognini (p), O Brändli (p), C Brombach (n), M Brutsche (p), L Burdet (p), M Frey (p), U Frey (pd), MW Gerbase (p), D Gold (e/c/p), E de Groot (c), W Karrer (p), R Keller (p), B Knöpfli (p), B Martin (pa), D Miedinger (o), U Neu (exp), L Nicod (p), M Pons (p), F Roche (c), T Rothe (p), E Russi (p), P Schmid-Grendelmeyer (a), A Schmidt-Trucksäss (pa), A Turk (p), J Schwartz (e), D. Stolz (p), P Straehl (exp), JM Tschopp (p), A von Eckardstein (cc), E Zemp Stutz (e).

**Scientific team at coordinating centers**: M Adam (e/g), E Boes (g), PO Bridevaux (p), D Carballo (c), E Corradi (e), I Curjuric (e), J Dratva (e), A Di Pasquale (s), L Grize (s), D Keidel (s), S Kriemler (pa), A Kumar (g), M Imboden (g), N Maire (s), A Mehta (e), F Meier (e), H Phuleria (exp), E Schaffner (s), GA Thun (g) A Ineichen (exp), M Ragettli (e), M Ritter (exp), T Schikowski (e), G Stern (pd), M Tarantino (s), M Tsai (e), M Wanner (pa)

(a) allergology, (c) cardiology, (cc) clinical chemistry, (e) epidemiology, (exp) exposure, (g) genetic and molecular biology, (m) meteorology, (n) nutrition, (o) occupational health, (p) pneumology, (pa) physical activity, (pd) pediatrics, (s) statistics

**Acknowledgements**
The study could not have been done without the help of the study participants, technical and administrative support and the medical teams and field workers at the local study sites.
Local fieldworkers : Aarau: S Brun, G Giger, M Sperisen, M Stahel, Basel: C Bürli, C Dahler, N Oertli, I Harreh, F Karrer, G Novicic, N Wyttenbacher, Davos: A Saner, P Senn, R Winzeler, Geneva: F Bonfils, B Blicharz, C Landolt, J Rochat, Lugano: S Boccia, E Gehrig, MT Mandia, G Solari, B Viscardi, Montana: AP Bieri, C Darioly, M Maire, Payerne: F Ding, P Danieli A Vonnez, Wald: D Bodmer, E Hochstrasser, R Kunz, C Meier, J Rakic, U Schafroth, A Walder.

**Administrative staff**: C Gabriel, R Gutknecht.
